# Supplementary material for: Saturation kinetics and specificity of transporters for L-arginine and asymmetric dimethylarginine (ADMA) at the blood-brain and blood-CSF barriers
Source: PLoS One. 2025 Jun 11;20(6):e0320034. doi: 10.1371/journal.pone.0320034 (PMC12157126; doi:10.1371/journal.pone.0320034)
Supplement: S1 File — (DOCX) [file pone.0320034.s001.docx]

**Supplementary file**

**Saturation kinetics and specificity of transporters for L-arginine and asymmetric dimethylarginine (ADMA) at the blood-brain and blood-CSF barriers.**

**Short title:** Characterisation of transporters for arginine and ADMA.

**Mehmet Fidanboylu^1^ and Sarah Ann Thomas^1*^**

^1^King’s College London, Institute of Pharmaceutical Science, Franklin-Wilkins Building, Stamford Street, London, SE1 9NH, UK

**Corresponding author:** [sarah.thomas@kcl.ac.uk](mailto:sarah.thomas@kcl.ac.uk)


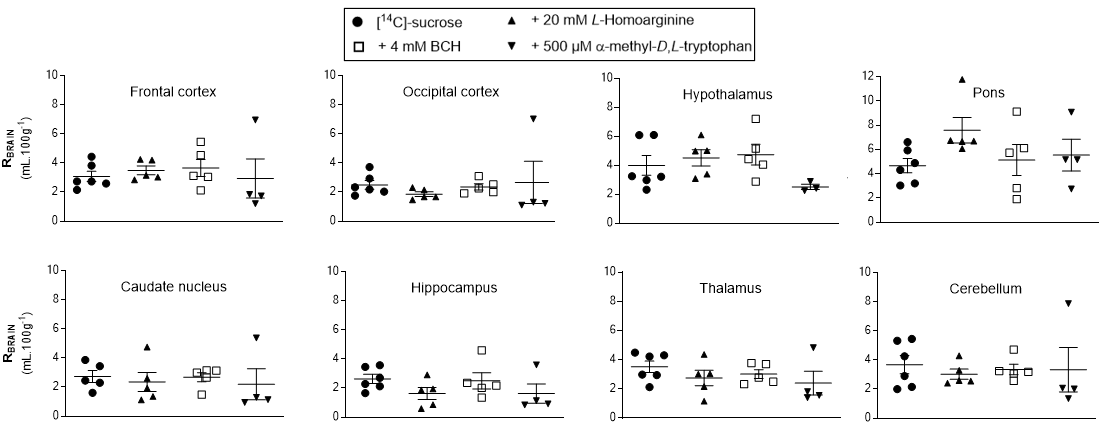


**S1 Fig: The effect of *L*-homoarginine, BCH and α-methyl-*D*,*L*-tryptophan on the regional brain uptake of [^14^C]-sucrose (10 minute perfusion; co-perfused with [^3^H]arginine).** Uptake is expressed as the percentage ratio of tissue to plasma (mL.100 g^-1^). Each bar represents the mean ± SEM of 3-6 animals Each marker represents one animal. One-way ANOVA with Dunnett's post-hoc test was used to compare means to control ([^14^C]-sucrose alone), with statistical significance taken as **p* <0.05, **p<0.01, ***p<0.001 (GraphPad Prism 10.2 for Windows).


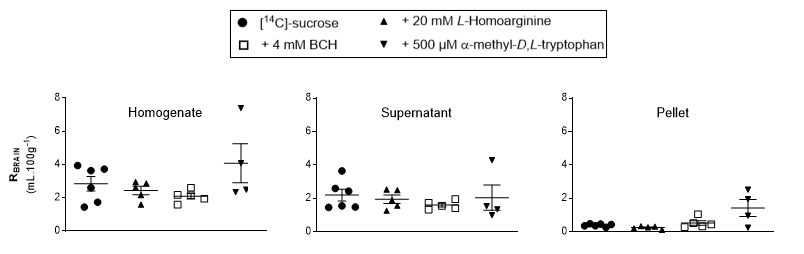


**S2 Fig: The effect of *L*-homoarginine, BCH and α-methyl-*D*,*L*-tryptophan uptake of [^14^C]-sucrose in capillary depletion samples (10 minute perfusion; co-perfused with [^3^H]arginine).** Uptake is expressed as the percentage ratio of tissue to plasma (mL.100 g^-1^). Each bar represents the mean ± SEM of 4-6 animals. Each marker represents one animal. One-way ANOVA with Dunnett's post-hoc test was used to compare means to control ([^14^C]-sucrose alone), with statistical significance taken as **p* <0.05, **p<0.01, ***p<0.001 (GraphPad Prism 10.2 for Windows).


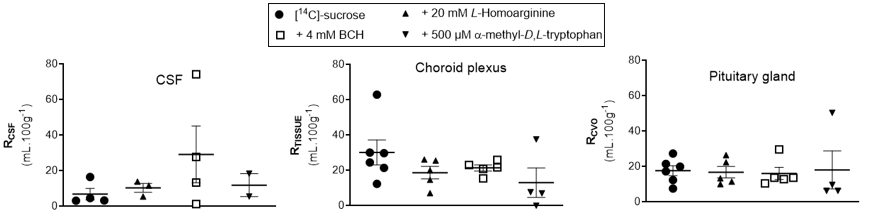


**S3 Fig: The effect of *L*-homoarginine, BCH and α-methyl-*D*,*L*-tryptophan on the uptake of [^14^C]-sucrose in CSF, choroid plexus and pituitary gland samples (10 minute perfusion; co-perfused with [^3^H]arginine).** Uptake is expressed as the percentage ratio of tissue to plasma (mL.100 g^-1^). Each bar represents the mean ± SEM of 4-6 animals, except the CSF samples for the *L*-homoarginine and the α-methyl-*D*,*L*-tryptophan inhibitor groups where n=3 and n=2, respectively. Each marker represents one animal. One-way ANOVA with Dunnett's post-hoc test was used to compare means to control ([^14^C]-sucrose alone), with statistical significance taken as **p* <0.05, **p<0.01, ***p<0.001 (GraphPad Prism 10.2 for Windows).

**
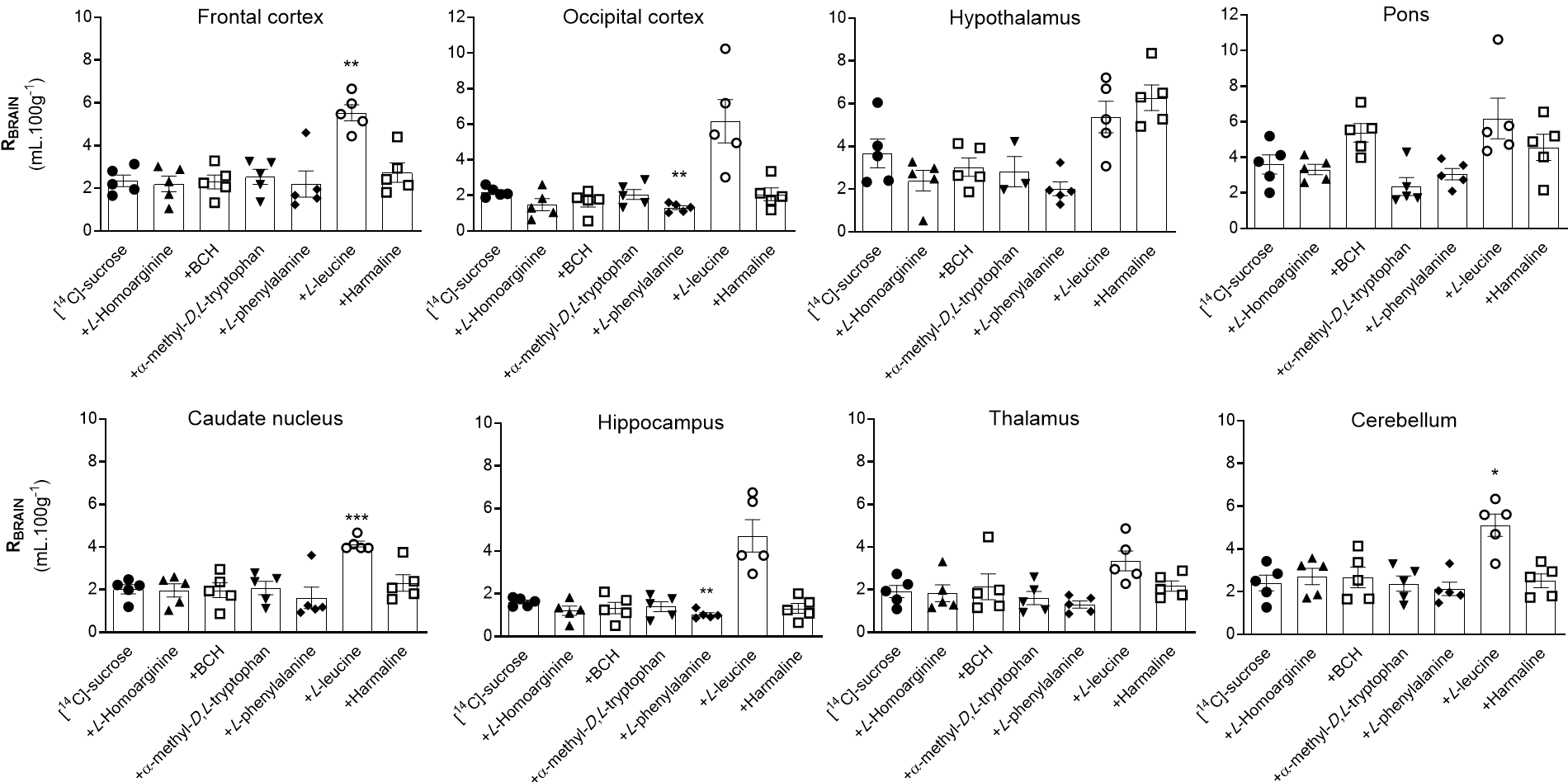
 S4 Fig: The effect of 20 mM *L*-homoarginine, 4 mM BCH, 500 μM a-methyl-*D*,*L*-tryptophan, 200 μM *L-*phenylalanine, 5 mM *L*-leucine and 2 mM harmaline on the regional brain uptake of [^14^C]-sucrose (10 minute perfusion; co-perfused with [^3^H]ADMA).** Uptake is expressed as the percentage ratio of tissue to plasma (mL.100 g^-1^). Perfusion time is 10 minutes. Each bar represents the mean ± SEM of 4-5 animals. Each marker represents one animal. Asterisks represent one-way ANOVA with Dunnett’s post-hoc tests comparing mean±SEM to control within each sample/region, **p* < 0.05, ***p* < 0.01, ****p* < 0.001 (GraphPad Prism 10.2 for Windows).

**
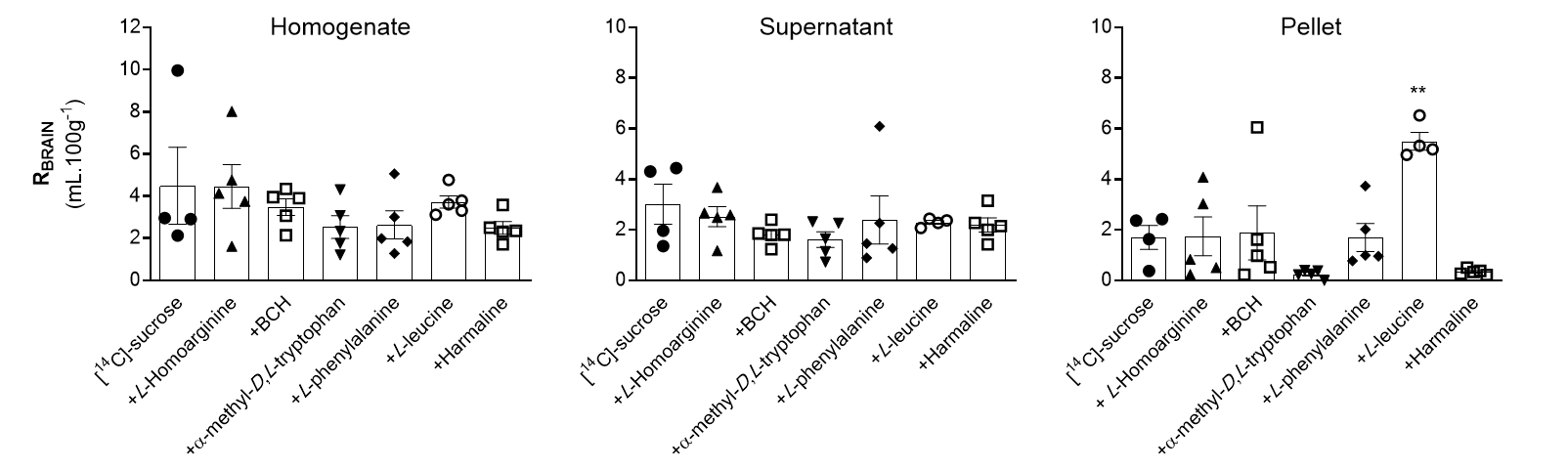
**

**S5 Fig: The effect of 20 mM *L*-homoarginine, 4 mM BCH, 500 μM a-methyl-*D*,*L*-tryptophan, 200 μM *L-*phenylalanine, 5 mM *L*-leucine and 2 mM harmaline on the distribution of [^14^C]-sucrose in capillary depletion samples (10 minute perfusion; co-perfused with [^3^H]-ADMA).** Uptake is expressed as the percentage ratio of tissue to plasma (mL.100 g^-1^) and is corrected for [^14^C]-sucrose. Each marker represents one animal. Perfusion time is 10 minutes. Each bar represents the mean ± SEM of 4-5 animals. Asterisks represent one-way ANOVA with Dunnett’s post-hoc tests comparing mean±SEM to control within each region/sample, **p* < 0.05, ***p* < 0.01 (GraphPad Prism 10.2 for Windows).

**
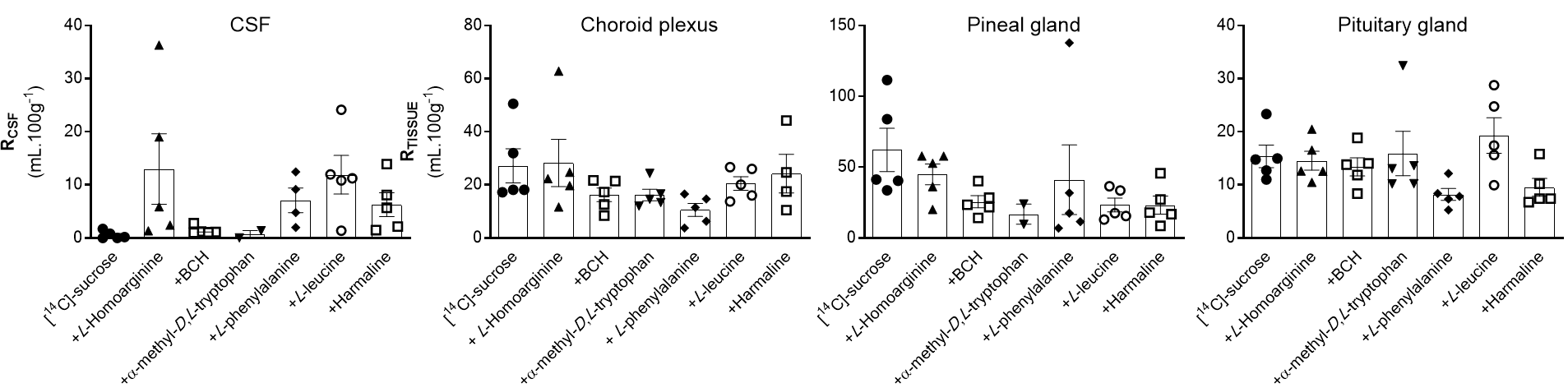
** **S6 Fig: The effect of 20 mM *L*-homoarginine, 4 mM BCH, 500 μM a-methyl-*D*,*L*-tryptophan, 200 μM *L-*phenylalanine, 5 mM *L*-leucine and 2 mM harmaline on the distribution of [^14^C]-sucrose in the CSF, choroid plexus and CVOs (10 minute perfusion; co-perfused with [^3^H]-ADMA).** Uptake is expressed as the percentage ratio of tissue to plasma (mL.100 g^-1^) and is corrected for [^14^C]-sucrose. Perfusion time is 10 minutes. Each bar represents the mean ± SEM of 4-5 animals except for the CSF and pineal samples where with the inhibitor group, α-methyl-*D,L*-tryptophan n = 2. Each marker represents one animal. Asterisks represent one-way ANOVA with Dunnett’s post-hoc tests comparing mean±SEM to control within each region/sample, **p* < 0.05, ***p* < 0.01 (GraphPad Prism 10.2 for Windows).


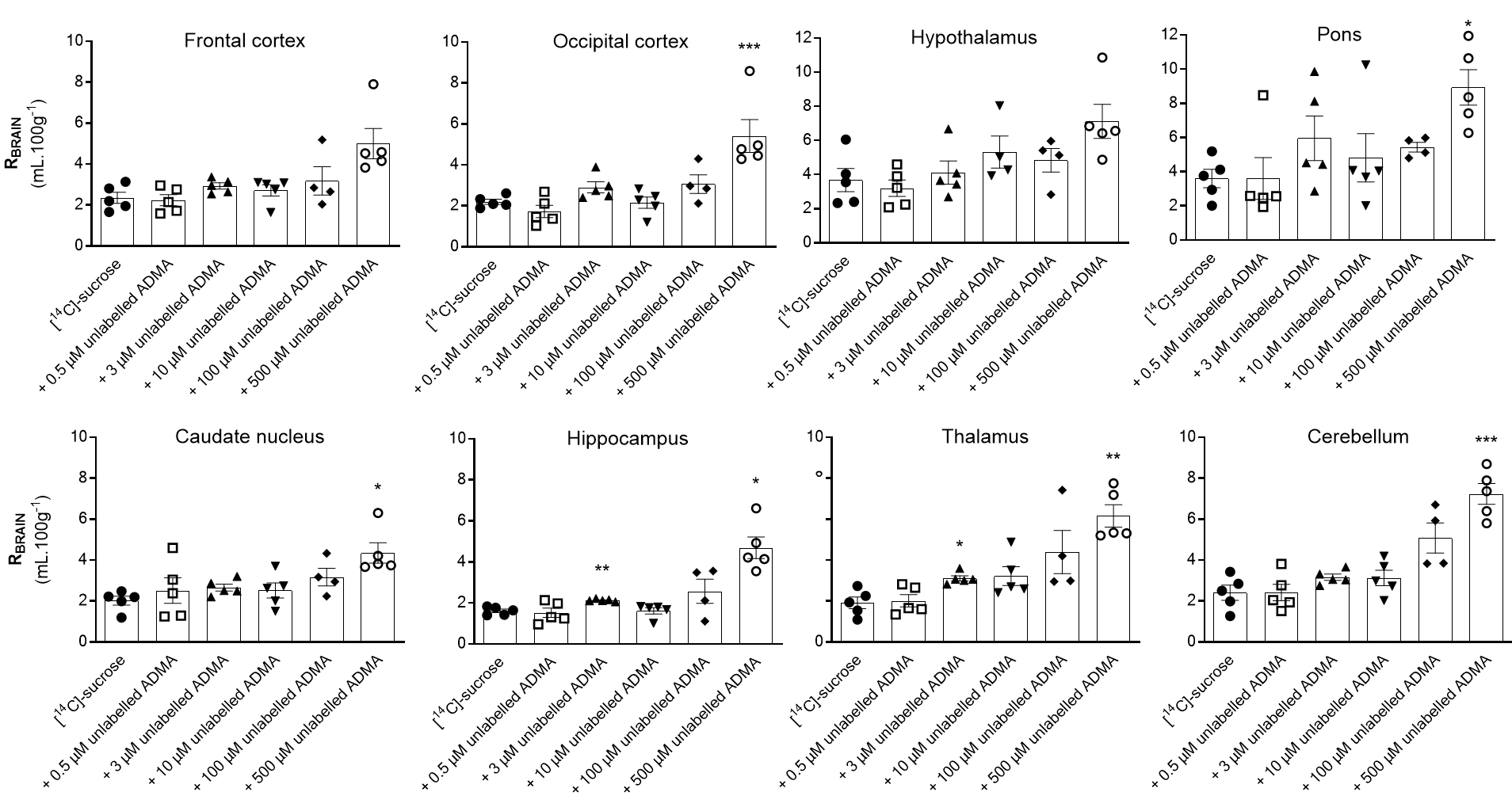


**S7 Fig: The effect of 0.5, 3, 10, 100 and 500 μM un-labelled ADMA on the uptake of [**^14^**C]-sucrose in the** **brain (10 minute perfusion; co-perfused with [^3^H]-ADMA).** Uptake is expressed as the percentage ratio of tissue to plasma (mL.100 g^-1^). Perfusion time is 10 minutes. Each bar represents the mean ± SEM of 4-5 animals. Each marker represents one animal. Asterisks represent one-way ANOVA with Dunnett’s post-hoc tests comparing mean±SEM to control, **p* < 0.05, ***p* < 0.01, ****p* < 0.001 (GraphPad Prism version 10.2.2 for windows).

**
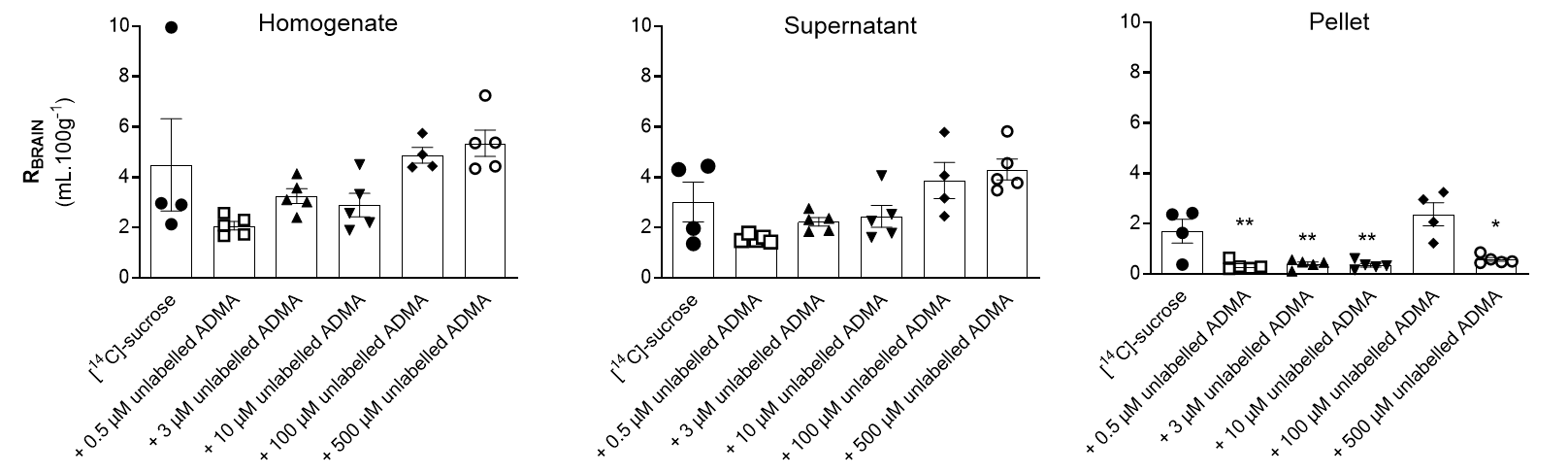
**

**S8 Fig: The effect of 0.5, 3, 10, 100 and 500 μM un-labelled ADMA on the uptake of [**^14^**C]-sucrose in the** **capillary depletion samples (10 minute perfusion; co-perfused with [^3^H]-ADMA).** Uptake is expressed as the percentage ratio of tissue to plasma (mL.100 g^-1^). Perfusion time is 10 minutes. Each bar represents the mean ± SEM of 4-5 animals. Each marker represents one animal. Asterisks represent one-way ANOVA with Dunnett’s post-hoc tests comparing mean±SEM to control, **p* < 0.05, ***p* < 0.01, ****p* < 0.001 (GraphPad Prism version 10.2.2 for windows). The [^14^C]-sucrose distribution into the pellet samples was statistically reduced by the presence of unlabelled ADMA at most concentrations, but was in the range achieved in other test groups where no statistical difference was obtained (S2 Fig). This suggests the membrane was intact in these samples.

**
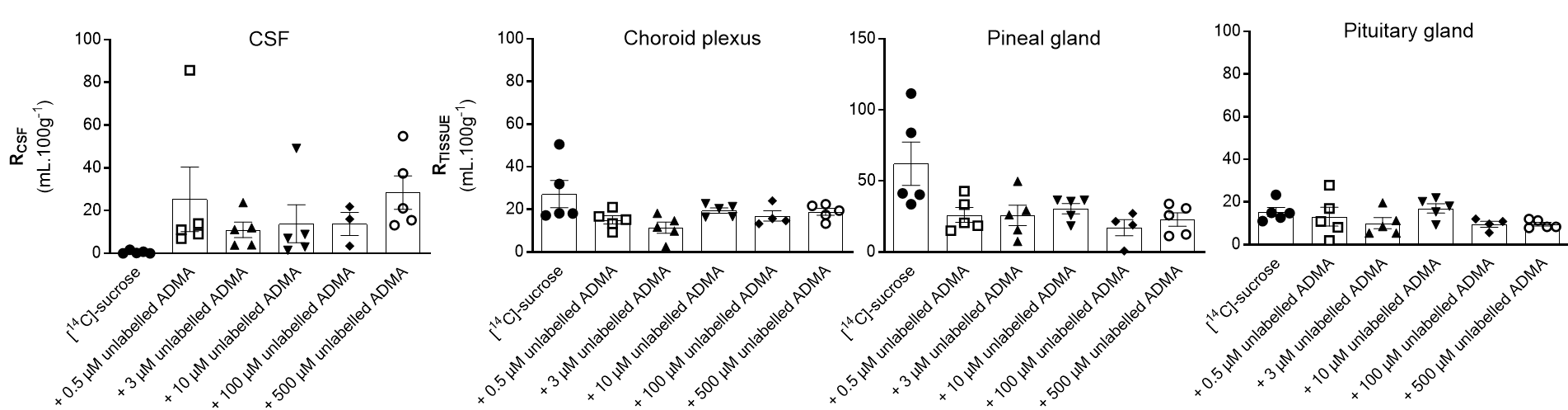
**

**S9 Fig: The effect of 0.5, 3, 10, 100 and 500 μM un-labelled ADMA on the uptake of [**^14^**C]-sucrose in the** **CSF and CVOs (10 minute perfusion; co-perfused with [^3^H]-ADMA).** Uptake is expressed as the percentage ratio of tissue to plasma (mL.100 g^-1^). Perfusion time is 10 minutes. Each bar represents the mean ± SEM of 3-5 animals. Each marker represents one animal. Asterisks represent one-way ANOVA with Dunnett’s post-hoc tests comparing mean±SEM to control, **p* < 0.05, ***p* < 0.01, ****p* < 0.001 (GraphPad Prism version 10.2.2 for windows).


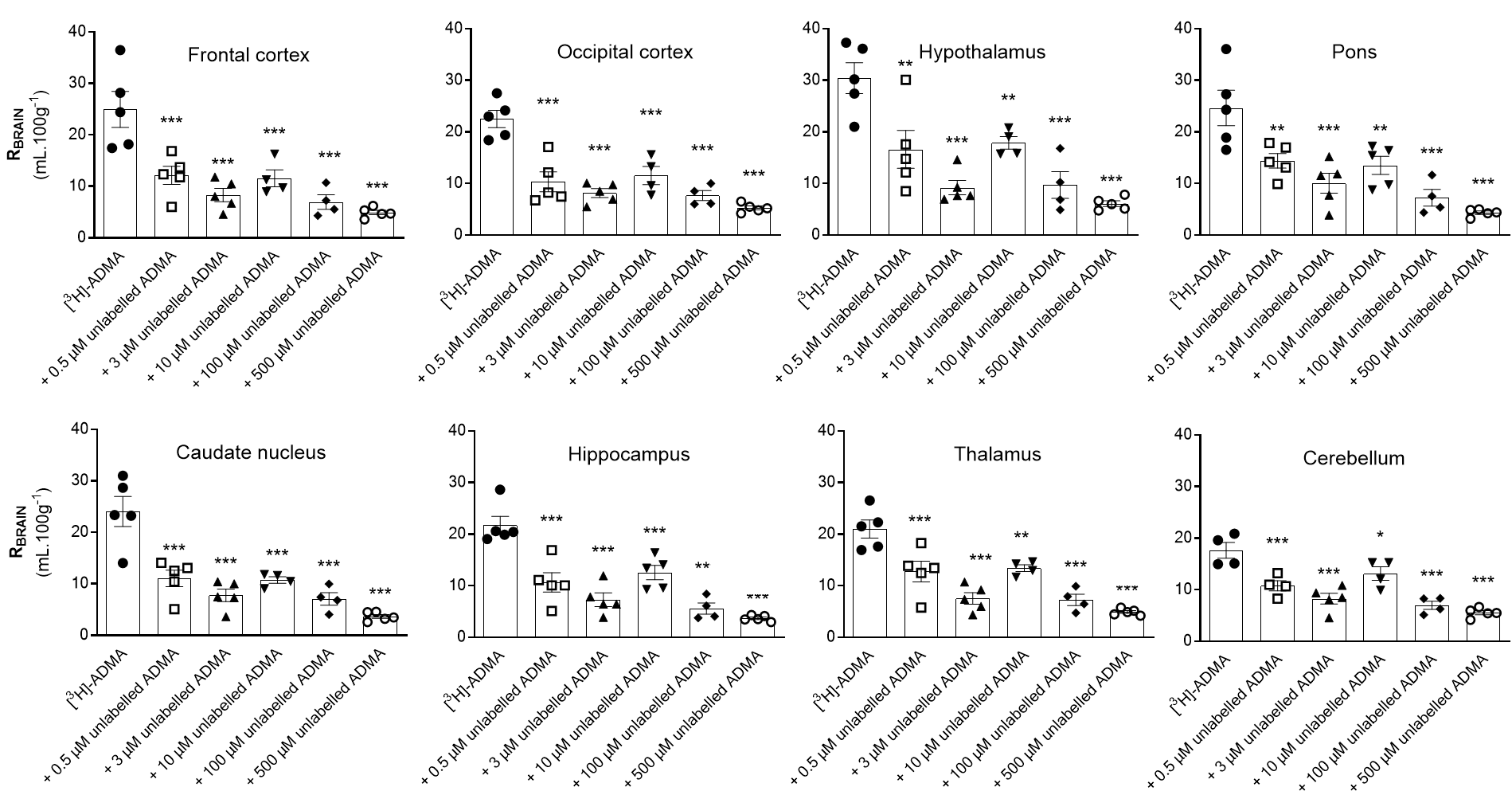


**S10 Fig: The effect of 0.5, 3, 10, 100 and 500 μM un-labelled ADMA on the uptake of [**^3^**H]-ADMA in the brain.** Uptake is expressed as the percentage ratio of tissue to plasma (mL.100 g^-1^) and is corrected for [^14^C]-sucrose (vascular space). Perfusion time is 10 minutes. Each marker represents one animal. Each bar represents the mean ± SEM of 4-5 animals. Asterisks represent one-way ANOVA with Dunnett’s post-hoc tests comparing mean±SEM to control, **p* < 0.05, ***p* < 0.01, ****p* < 0.001 (GraphPad Prism 6.0 for Mac).


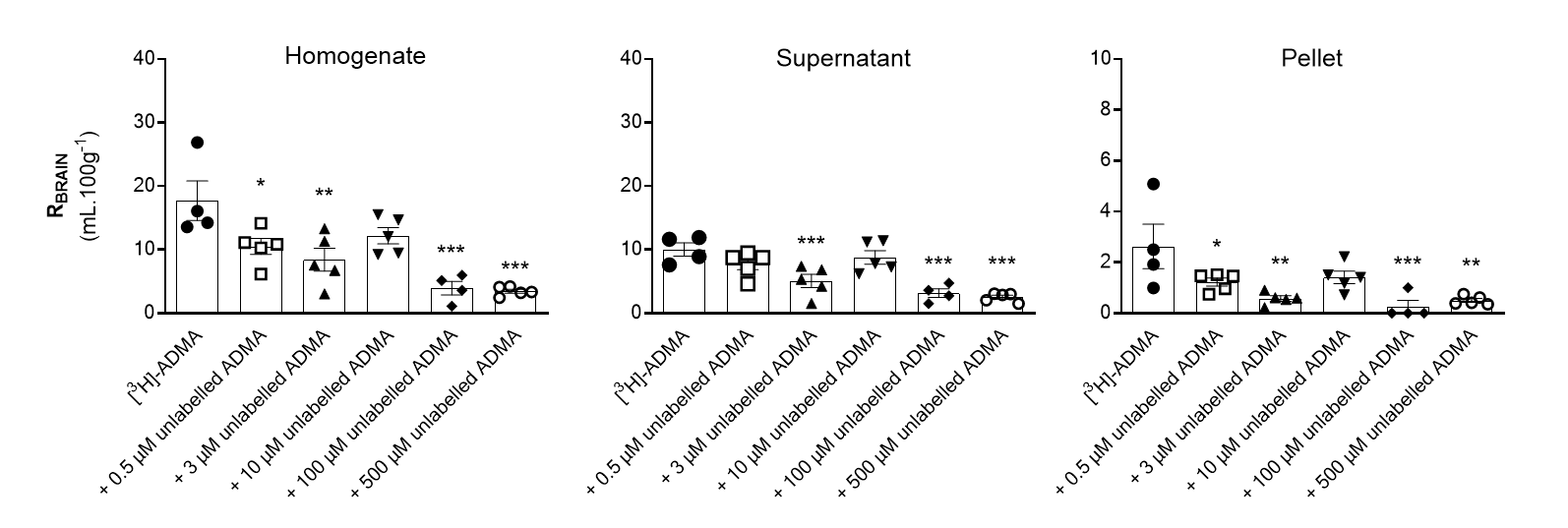
**S11 Fig: The effect of 0.5, 3, 10, 100 and 500 μM un-labelled ADMA on the distribution of [^3^H]-ADMA in capillary depletion samples.** Uptake is expressed as the percentage ratio of tissue to plasma (mL.100 g^-1^) and is corrected for [^14^C]-sucrose. Each marker represents one animal. Each bar represents the mean ± SEM of 4-5 animals. Asterisks represent one-way ANOVA with Dunnett’s post-hoc tests comparing mean±SEM to control, ns = p>0.05, **p* < 0.05, ***p* < 0.01, ****p* < 0.001 (GraphPad Prism version 10.2.2 for windows).


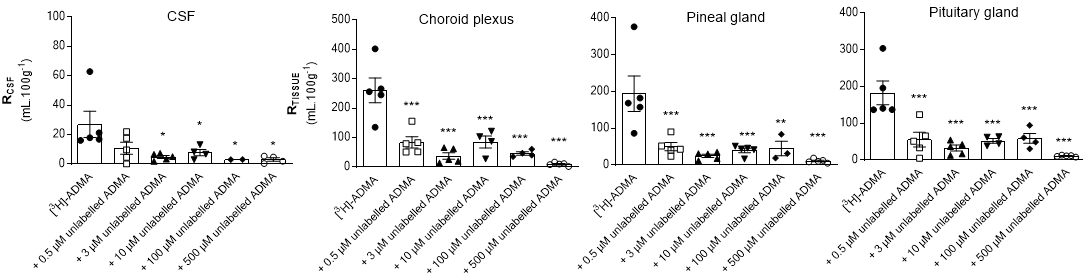


**S12 Fig: The effect of 0.5, 3, 10, 100 and 500 μM unlabelled ADMA on the distribution of [**^3^**H]-ADMA in the CSF and CVOs.** Uptake is expressed as the percentage ratio of tissue or CSF to plasma (mL.100 g^-1^) and is corrected for [^14^C]-sucrose. Each marker represents one animal. Each bar represents the mean ± SEM. n= 4-5 mice (for the CSF samples except at 100 μM where it was 2 mice), 4-5 mice (for the choroid plexus samples), 5 mice (for the pineal gland samples except at 100 μM where it was 3 mice) and 4-5 mice (for the pituitary gland samples) at each of the 6 ADMA concentrations. (GraphPad Prism version 10.2.2 for windows). Asterisks represent one-way ANOVA with Dunnett’s post-hoc tests comparing mean±SEM to control, ns = p>0.05, **p* < 0.05, ***p* < 0.01, ****p* < 0.001.

**
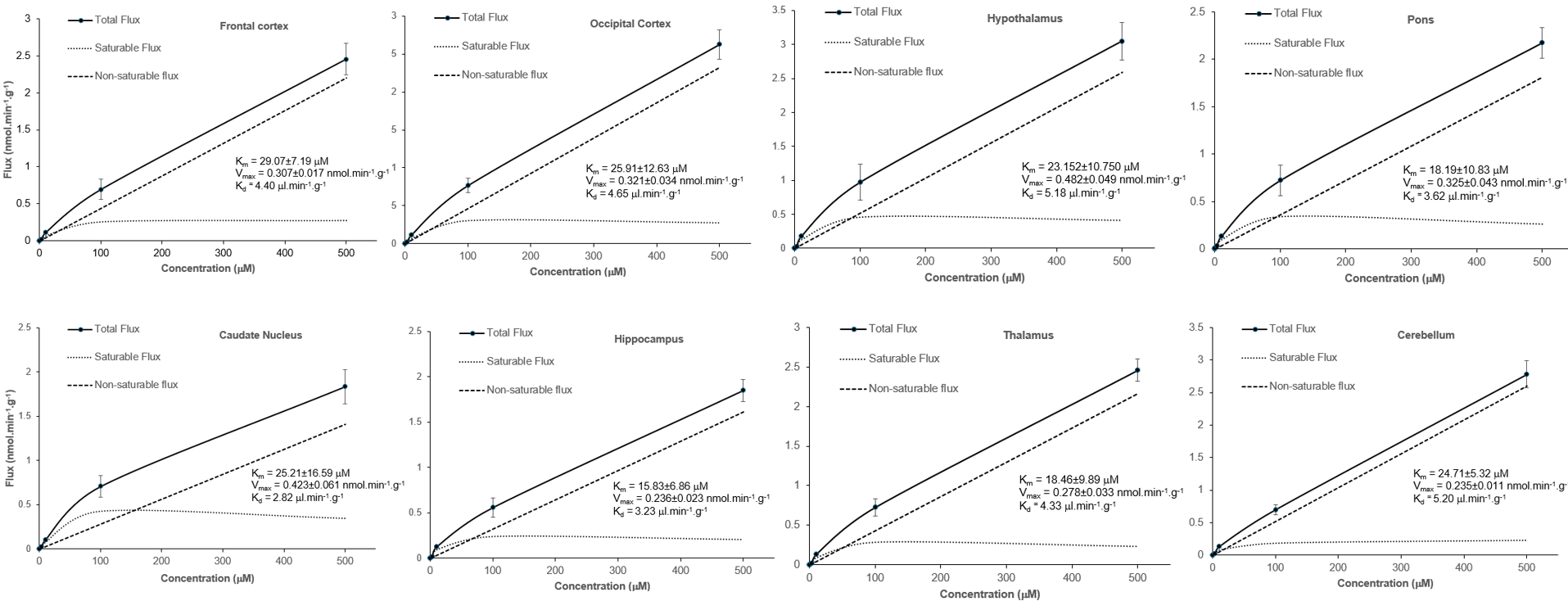
**

**S13 Fig:** **The contributions of the saturable and non-saturable components to total brain flux of [^3^H]ADMA are plotted against the unlabelled ADMA concentration**. The measured values are the mean ±SEM for 4-5 mice at each of the 6 ADMA concentrations and has been [^14^C]-sucrose corrected. The lower lines show the contributions of the saturable and non-saturable components to total influx. The K_d_ value was calculated from linear regression analysis of the total flux at the highest concentrations. The K_m_ and V_max_ were calculated by Michaelis-Menten kinetic analysis of the saturable flux (mean values were used). Analyses were performed using GraphPad Prism version10. Unlabelled ADMA concentrations of 500 μM did statistically increase the distribution of [^14^C]-sucrose into the occipital cortex, caudate nucleus, hippocampus, thalamus, pons and cerebellum and the values measured were at the upper range of [^14^C]-sucrose values achieved at the lower concentrations of unlabelled ADMA (i.e. ≤100 μM unlabelled ADMA; S7 Fig). As this does suggest loss of BBB integrity in these regions, we only interpreted the kinetic characteristics of [^3^H]-ADMA in the other regions where the BBB remained statistically intact (i.e. frontal cortex, hypothalamus). However, all regions are presented here for comparison.


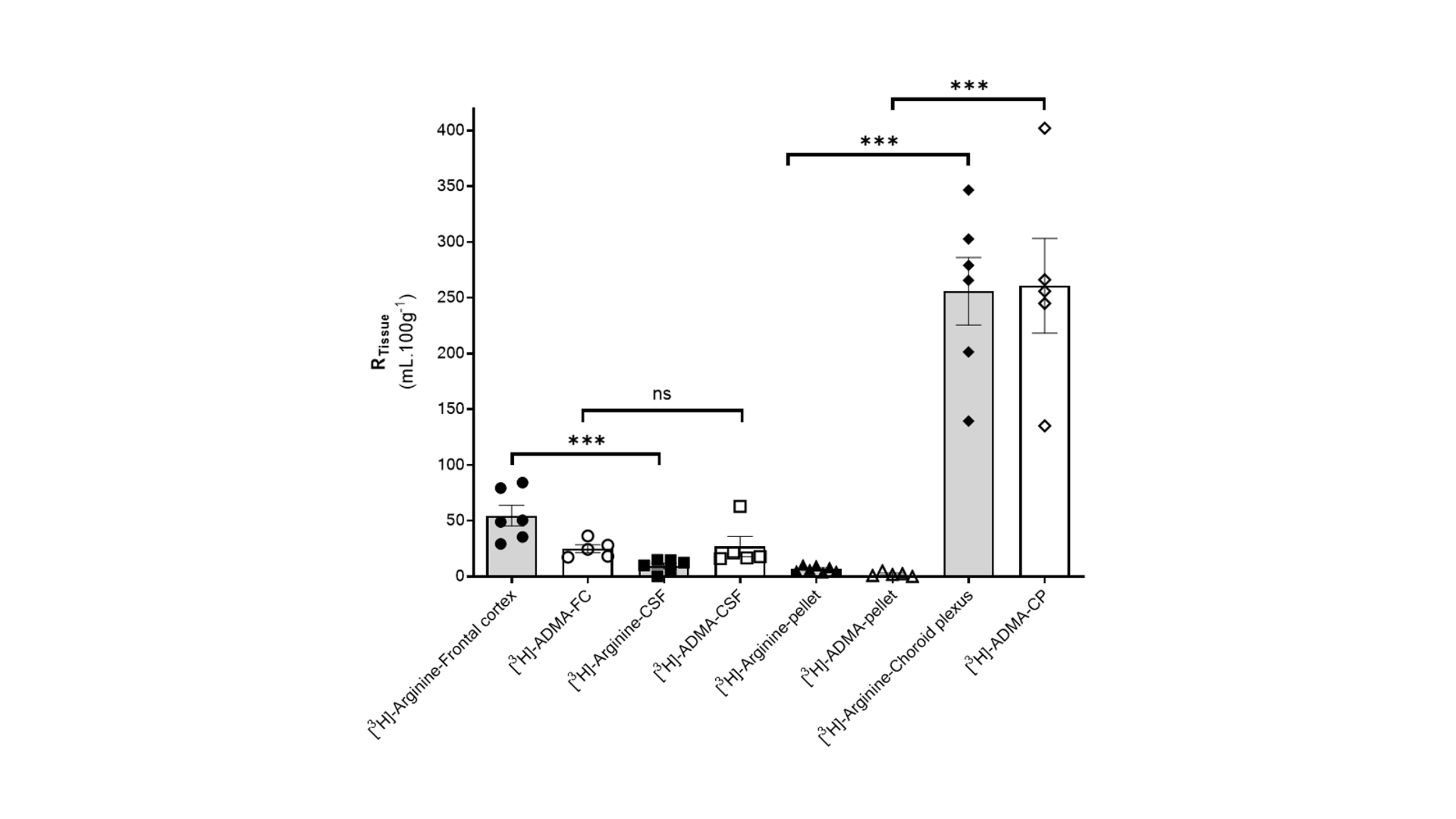
 **S14 Fig: Comparison of [^3^H]-arginine and [^3^H]-ADMA distribution into frontal cortex (FC), CSF, capillary endothelial cell enriched pellet and choroid plexus (CP).** Uptake is expressed as the percentage ratio of tissue to plasma (mL.100 g^-1^) and is corrected for [^14^C]-sucrose. Perfusion time is 10 minutes. Each bar represents the mean ± SEM of 5-7 animals. Each marker represents one animal. Asterisks represent unpaired t-tests **p* < 0.05, ***p* < 0.01, ****p* < 0.001 (GraphPad Prism 10.0 for Windows).


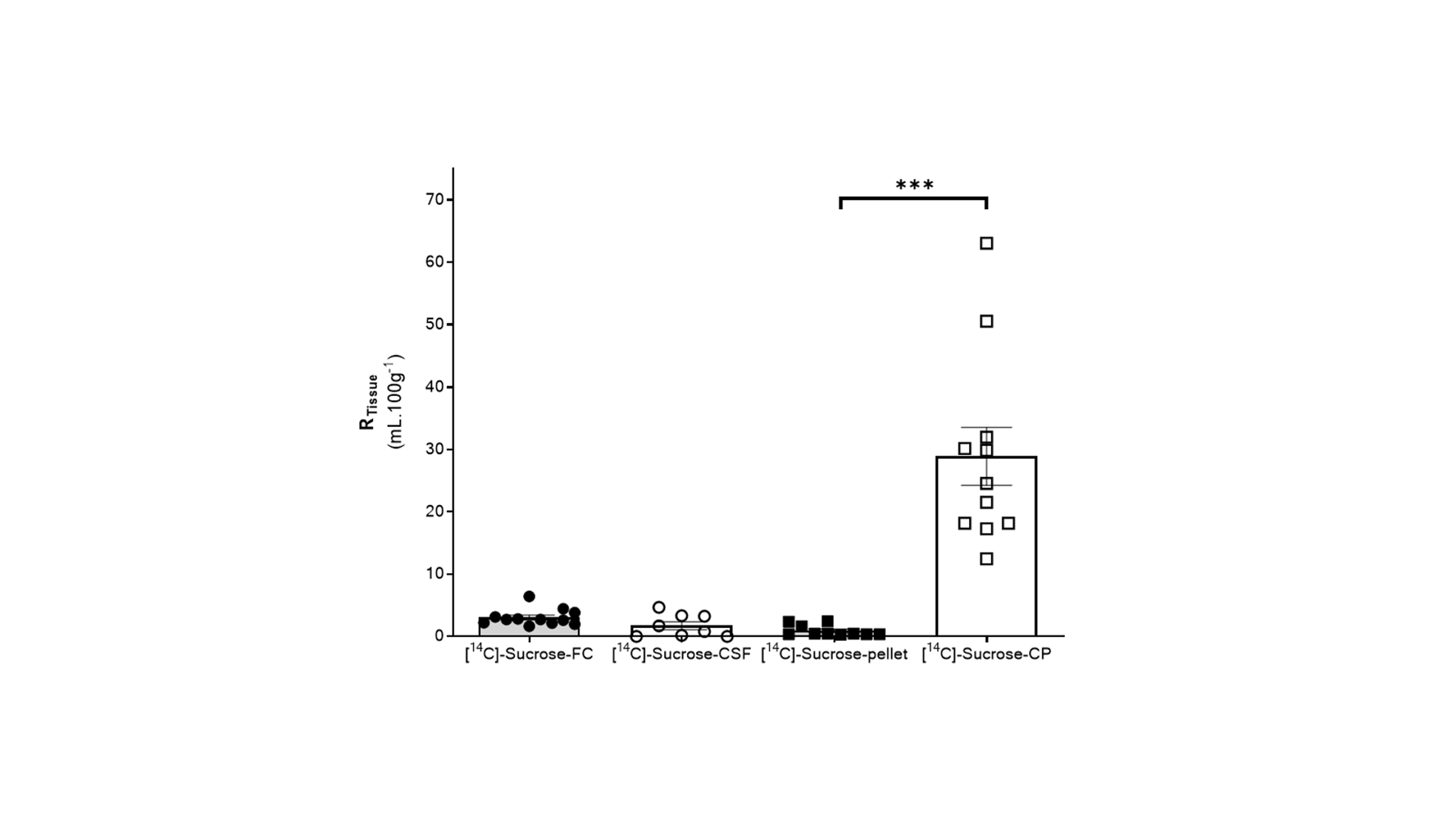


**S15 Fig: Comparison of [^14^C]-sucrose into frontal cortex (FC), CSF, capillary endothelial cell enriched pellet and choroid plexus (CP).** Uptake is expressed as the percentage ratio of tissue to plasma (mL.100 g^-1^). Perfusion time is 10 minutes. Each bar represents the mean ± SEM of 8-11 animals. Each marker represents one animal. Asterisks represent unpaired t-tests **p* < 0.05, ***p* < 0.01, ****p* < 0.001 (GraphPad Prism 10.0 for Windows).

**S1 Table:** **The amino acid transport systems studied, the transporter inhibitors, the inhibitor concentration utilised, transporter protein inhibited and their respective gene codes according to the human genome organisation (HUGO).** A complex of different proteins may mediate a distinct system activity.

| **System** | **Inhibitor and molar concentration** | **Transporter protein inhibited** | **Gene Codes**  Solute carrier family (SLC) *number* member A *number* | **References** |
| --- | --- | --- | --- | --- |
| y^+^ | *L*-homoarginine  (20 mM) | CAT-1 | SLC7A1 | [1]  [2] |
| B^0,+^ | BCH  (4 mM) | ATB^0,+^,  LAT-1 | SLC6A14  SLC7A5 | [3][4][5][6] |
| B^0,+^ | α-methyl-D,L-tryptophan  (500 μM) | ATB^0,+^, | SLC6A14 | [7,8] |
| L | *L*-phenylalanine  (200 μM) | LAT-1 | SLC7A5 | [9] |
| y^+^L  B^0,+^  L | *L*-leucine  (5 mM) | y^+^LAT-1 or y^+^LAT -2  ATB^0,+^,  LAT-1 | SLC7A7 or SLC7A6  SLC6A14  SLC7A5 | [10][11] [12] |
| b^0,+^ | Harmaline  (2 mM) | b^0,+^ AT / rBAT | SLC7A9 / SLC3A1 heteromeric complex | [13] |

**S2 Table: Results obtained from specific transport-inhibition studies for [^3^H]-ADMA uptake into the brain.** Uptake is expressed as the percentage ratio of tissue to plasma (mL.100 g^-1^) and is corrected for [^14^C]-sucrose (vascular space). Perfusion time is 10 minutes. One-way ANOVA with Dunnett's post-hoc test was used to compare means to control ([^3^H]-ADMA alone), with statistical significance taken as *p* < 0.05. n.s. = not significant. The percentage inhibition is reported where appropriate. Each value has been obtained from an n of 5 experiments except where stated. **^#^**The [^14^C]-sucrose (vascular space) in that brain region was significantly affected by the presence of the inhibitor (i.e. *L-*phenylalanine or *L-*leucine).

| **TISSUE** | **R_Brain_**  **(ml.100g^-1^)** | **R_Brain_**  **(ml.100g^-1^)** | **% inhibition** | ***p*** |
| --- | --- | --- | --- | --- |
| **FRONTAL CORTEX** |  |  |  |  |
| [^3^H]-ADMA alone | 24.94±3.50 |  |  |  |
| + 20 mM *L*-homoarginine |  | 1.06±1.02 | 95.7 | p<0.001 |
| + 4 mM BCH |  | 15.45±1.98 | **-** | n.s. |
| + 500 μM α-methyl-*D*,*L*-tryptophan |  | 12.97±3.94 | 48.0 | p<0.05 |
| 200 μM *L*-phenylalanine |  | 10.8±1.35 | 56.7 | p<0.01 |
| 5 mM *L*-leucine |  | 8.78±1.56 | 64.8**^#^** | p<0.001 |
| 2 mM Harmaline |  | 13.68±2.95 | 45.1 | p<0.05 |
| **CAUDATE NUCLEUS** |  |  |  |  |
| [^3^H]-ADMA alone | 24.07±2.92 |  |  |  |
| + 20 mM *L*-homoarginine |  | 0.18±0.13 | 99.2 | p<0.001 |
| + 4 mM BCH |  | 15.04±2.31 | 37.5 | p<0.05 |
| + 500 μM α-methyl-*D*,*L*-tryptophan |  | 12.24±3.14 | 49.1 | p<0.01 |
| + 200 μM *L*-phenylalanine |  | 9.39±0.96 | 61.0 | p<0.001 |
| + 5 mM *L*-leucine |  | 7.38±0.99 | 69.3**^#^** | P<0.001 |
| + 2 mM Harmaline |  | 11.94±2.51 | 50.4 | P<0.01 |
| **OCCIPITAL CORTEX** |  |  |  |  |
| [^3^H]-ADMA alone | 22.52±1.65 |  |  |  |
| + 20 mM *L*-homoarginine |  | 0.49±0.40 | 97.8 | p<0.001 |
| + 4 mM BCH |  | 14.82±2.41 | 34.2 | p<0.05 |
| + 500 μM α-methyl-*D*,*L*-tryptophan |  | 12.28±2.41 | 45.5 | p<0.001 |
| + 200 μM *L*-phenylalanine |  | 8.86±0.59 | 60.7**^#^** | p<0.001 |
| + 5 mM *L*-leucine |  | 6.69±1.50 | 70.3 | P<0.001 |
| + 2 mM Harmaline |  | 10.65±1.49 | 52.7 | P<0.001 |
| **HIPPOCAMPUS** |  |  |  |  |
| [^3^H]-ADMA alone | 21.74±1.75 |  |  |  |
| + 20 mM *L*-homoarginine |  | 0.20±0.13 | 99.1 | p<0.001 |
| + 4 mM BCH |  | 13.79±2.21 | 36.6 | p<0.05 |
| + 500 μM α-methyl-*D*,*L*-tryptophan |  | 14.17±3.69 | - | n.s. |
| + 200 μM *L*-phenylalanine |  | 10.05±1.32 | 53.8**^#^** | p<0.01 |
| + 5 mM *L*-leucine |  | 7.45±1.24 | 65.7 | P<0.001 |
| + 2 mM Harmaline |  | 11.45±1.97 | 47.3 | P<0.01 |
| **HYPOTHALAMUS** |  |  |  |  |
| [^3^H]-ADMA alone | 30.42 ± 2.98 |  |  |  |
| + 20 mM *L*-homoarginine |  | 1.54±1.12 | 94.9 | p<0.001 |
| + 4 mM BCH |  | 19.23±2.51  (n=4) | 36.8 | p<0.05 |
| + 500 μM α-methyl-*D*,*L*-tryptophan |  | 19.34±6.28  (n=3) | - | n.s. |
| + 200 μM *L*-phenylalanine |  | 12.38±0.89 | 59.3 | p<0.001 |
| + 5 mM *L*-leucine |  | 15.81±1.84 | 48.0 | P<0.01 |
| + 2 mM Harmaline |  | 18.56±2.79 | 39.0 | P<0.05 |
| **THALAMUS** |  |  |  |  |
| [^3^H]-ADMA alone | 21.09±1.74 |  |  |  |
| + 20 mM *L*-homoarginine |  | 1.81±0.71 | 91.4 | p<0.001 |
| + 4 mM BCH |  | 15.82±2.69 | - | n.s. |
| + 500 μM α-methyl-*D*,*L*-tryptophan |  | 17.12±6.91  (n=4) | - | n.s. |
| + 200 μM *L*-phenylalanine |  | 10.54±0.65 | 49.8 | p<0.05 |
| + 5 mM *L*-leucine |  | 10.31±1.06 | 50.9 | P<0.05 |
| + 2 mM Harmaline |  | 13.29±2.03 | 36.7 | n.s. |
| **PONS** |  |  |  |  |
| [^3^H]-ADMA alone | 24.64±3.44 |  |  |  |
| + 20 mM *L*-homoarginine |  | 2.36±1.06 | 90.4 | p<0.001 |
| + 4 mM BCH |  | 19.44±2.80 | - | n.s. |
| + 500 μM α-methyl-*D*,*L*-tryptophan |  | 20.83±9.92  (n=4) | - | n.s. |
| + 200 μM *L*-phenylalanine |  | 11.71±1.35 | - | n.s. |
| + 5 mM *L*-leucine |  | 11.38±1.67 | - | n.s. |
| + 2 mM Harmaline |  | 11.57±1.29 | - | n.s. |
| **CEREBELLUM** |  |  |  |  |
| [^3^H]-ADMA alone | 17.65±1.52  (n=4) |  |  |  |
| + 20 mM *L*-homoarginine |  | 0.37±0.19 | 97.9 | p<0.001 |
| + 4 mM BCH |  | 14.29±1.56 | - | n.s. |
| + 500 μM α-methyl-*D*,*L*-tryptophan |  | 13.55±3.09 | - | n.s. |
| + 200 μM *L*-phenylalanine |  | 9.84±1.27 | - | n.s |
| + 5 mM *L*-leucine |  | 8.66±1.07 | -**^#^** | n.s. |
| + 2 mM Harmaline |  | 12.02±2.52 | **-** | n.s. |

**S3 Table: Results obtained from specific transport-inhibition studies for [^3^H]-ADMA in capillary depletion samples.** Uptake is expressed as the percentage ratio of tissue to plasma (mL.100 g^-1^) and is corrected for [^14^C]-sucrose. One-way ANOVA with Dunnett's post-hoc test was used to compare means to control ([^3^H]-ADMA alone), with statistical significance taken as *p* < 0.05. n = number of experiments. The percentage inhibition is reported where appropriate. Each value has been obtained from an n of 5 experiments except where stated. n.s. = not significant. #The [^14^C]-sucrose value in that compartment was significantly increased by the presence of the inhibitor, *L-*leucine.

| **TISSUE** | **R_Brain_**  **(ml.100g^-1^)** | **R_Brain_**  **(ml.100g^-1^)** | **% inhibition** | ***p*** |
| --- | --- | --- | --- | --- |
| **HOMOGENATE** |  |  |  |  |
| [^3^H]-ADMA alone | 17.70±3.10  (n=4) |  |  |  |
| + 20 mM *L*-homoarginine |  | 3.74±3.52 | 78.9 | p<0.001 |
| + 4 mM BCH |  | 11.22±1.22 | - | n.s. |
| + 500 μM α-methyl-*D*,*L*-tryptophan |  | 15.42±4.47  (n=5) | - | n.s. |
| + 200 μM *L*-phenylalanine |  | 6.85±0.55 | 61.3 | p<0.05 |
| + 5 mM *L*-leucine |  | 6.43±1.23 | 63.7 | p<0.05 |
| + 2 mM Harmaline |  | 11.99±2.044 | **-** | n.s. |
| **SUPERNATANT** |  |  |  |  |
| [^3^H]-ADMA alone | 10.03 ± 1.06  (n=4) |  |  |  |
| + 20 mM *L*-homoarginine |  | 3.43±1.95 | 65.8 | p<0.05 |
| + 4 mM BCH |  | 8.48±1.22 | - | n.s. |
| + 500 μM α-methyl-*D*,*L*-tryptophan |  | 9.32±2.33 | - | n.s. |
| + 200 μM *L*-phenylalanine |  | 5.01±0.52 | - | n.s. |
| + 5 mM *L*-leucine |  | 7.30±0.86  (n=4) | - | n.s. |
| + 2 mM Harmaline |  | 9.08±1.69 | **-** | n.s. |
| **PELLET** |  |  |  |  |
| [^3^H]-ADMA alone | 2.62±0.88  (n=4) |  |  |  |
| + 20 mM *L*-homoarginine |  | 2.32±1.65 | - | n.s. |
| + 4 mM BCH |  | 0.94±0.26 | - | n.s. |
| + 500 μM α-methyl-*D*,*L*-tryptophan |  | 2.14±0.93 | - | n.s. |
| + 200 μM *L*-phenylalanine |  | 0.34±0.12 | - | n.s. |
| + 5 mM *L*-leucine |  | 0±0**^#^**  (n=4) | - | n.s. |
| + 2 mM Harmaline |  | 1.72±0.27 | **-** | n.s. |

**S4 Table: Results obtained from specific transport-inhibition studies for [^3^H]-ADMA in the CSF, choroid plexus and CVO’s.** Uptake is expressed as the percentage ratio of tissue to plasma (mL.100 g^-1^) and is corrected for [^14^C]-sucrose. One-way ANOVA with Dunnett's post-hoc test was used to compare means to control ([^3^H]-ADMA alone), with statistical significance taken as *p* < 0.05. n = number of experiments. Each value has been obtained from an n of 5 experiments except where stated. n.s. = not significant.

| **TISSUE** | **R_Brain_**  **(ml.100g^-1^)** | **R_Brain_**  **(ml.100g^-1^)** | **% inhibition** | ***p*** |
| --- | --- | --- | --- | --- |
| **CSF** |  |  |  |  |
| [^3^H]-ADMA alone | 26.72±8.84 |  |  |  |
| + 20 mM *L*-homoarginine |  | 2.45±0.52 | - | n.s. |
| + 4 mM BCH |  | 7.00±2.41 | - | n.s. |
| + 500 μM α-methyl-*D*,*L*-tryptophan |  | 13.91±13.84  (n=2) | - | n.s. |
| + 200 μM *L*-phenylalanine |  | 9.34±5.97  (n=4) | - | n.s. |
| + 5 mM *L*-leucine |  | 12.62±3.18 | - | n.s. |
| + 2 mM Harmaline |  | 8.92±1.93 | **-** | n.s. |
| **PINEAL GLAND** |  |  |  |  |
| [^3^H]-ADMA alone | 194.1±48.46 |  |  |  |
| + 20 mM *L*-homoarginine |  | 12.43±6.87 | 93.6 | p<0.001 |
| + 4 mM BCH |  | 66.69±25.76 | 65.6 | p<0.01 |
| + 500 μM α-methyl-*D*,*L*-tryptophan |  | 37.57±16.10  (n=2) | 80.6 | p<0.01 |
| + 200 μM *L*-phenylalanine |  | 42.28±11.95 | 78.2 | p<0.001 |
| + 5 mM *L*-leucine |  | 43.02±7.99 | 77.8 | p<0.001 |
| + 2 mM Harmaline |  | 54.61±14.48  (n=4) | 71.9 | p<0.01 |
| **CHOROID PLEXUS** |  |  |  |  |
| [^3^H]-ADMA alone | 260.9±42.45 |  |  |  |
| + 20 mM *L*-homoarginine |  | 30.73±22.20 | 88.2 | p<0.001 |
| + 4 mM BCH |  | 130.5±23.12 | 50.0 | p<0.05 |
| + 500 μM α-methyl-*D*,*L*-tryptophan |  | 184.1±48.8 | - | n.s. |
| + 200 μM *L*-phenylalanine |  | 87.79±17.33 | 66.4 | p<0.01 |
| + 5 mM *L*-leucine |  | 70.22±4.095 | 73.1 | p<0.001 |
| + 2 mM Harmaline |  | 85.53±21.96  (n=4) | 67.2 | p<0.01 |
| **PITUITARY GLAND** |  |  |  |  |
| [^3^H]-ADMA alone | 182.9±32.28 |  |  |  |
| + 20 mM *L*-homoarginine |  | 8.57±3.99 | 95.3 | p<0.001 |
| + 4 mM BCH |  | 70.42±11.35 | 61.5 | p<0.01 |
| + 500 μM α-methyl-*D*,*L*-tryptophan |  | 134.1±43.38  (n=4) | - | n.s. |
| + 200 μM *L*-phenylalanine |  | 37.03±7.45 | 79.8 | p<0.001 |
| + 5 mM *L*-leucine |  | 53.73±16.95 | 70.6 | p<0.001 |
| + 2 mM Harmaline |  | 54.75±1.88 | 70.1 | p<0.001 |

**S5 Table: The kinetic constants for [^3^H]-ADMA influx into the different brain regions, capillary depletion samples and the CSF.** The K_m_ and V_max_ values were calculated using the saturable flux, which was determined from the total flux by correcting for non-saturable flux (K_d_ × concentration). Except those for CSF which were calculated from the total flux as there was no non-saturable component. **^#^**The [^14^C]-sucrose (vascular space) in the majority of brain region (except the frontal cortex and hypothalamus) was significantly increased by the presence of the 500 μM unlabelled ADMA. Despite this the kinetic constants (K_m_) values calculated in the each of the brain regions were not statistically significantly different to each other (One-Way ANOVA followed by Tukey’s multiple comparison test). ^†^The [^14^C]-sucrose distribution into the pellet samples was statistically reduced by the presence of unlabelled ADMA at all concentrations (except 100 μM ADMA where there was no difference) but was in the range achieved in other test groups where no statistical difference was obtained (S2 Fig). This suggests the membrane was intact in these samples.

| **Tissue** | **K_m_**  **(μM)** | **V_max_**  **(nmol.min^-1^.g^-1^)** | **K_d_**  **(μl min^-1^g^-1^)** |
| --- | --- | --- | --- |
| **Brain Regions** |  |  |  |
| Frontal Cortex | 29.07±7.19 | 0.307±0.017 | 4.40 |
| Caudate nucleus**^#^** | 25.21±16.59 | 0.423±0.061 | 2.82 |
| Occipital cortex**^#^** | 25.91±12.63 | 0.321±0.034 | 4.65 |
| Hippocampus**^#^** | 15.83±6.86 | 0.24±0.02 | 3.23 |
| Hypothalamus | 23.15±10.75 | 0.482±0.049 | 5.18 |
| Thalamus**^#^** | 18.46±9.89 | 0.278±0.033 | 4.33 |
| Pons**^#^** | 18.19±10.83 | 0.325±0.043 | 3.62 |
| Cerebellum**^#^** | 24.71±5.32 | 0.235±0.011 | 5.20 |
| **Capillary depletion Samples** |  |  |  |
| Homogenate | 13.59±5.27 | 0.155±0.013 | 3.34 |
| Supernatant | 68.52±44.41 | 0.208±0.036 | 2.35 |
| Pellet ^†^ | 33.97±16.83 | 0.071±0.008 | 0.38 |
| **CSF** |  |  |  |
| CSF | 30.59±25.41 | 2.067±0.38 | - |

**References**

1. White MF. The transport of cationic amino acids across the plasma membrane of mammalian cells. Biochimica et Biophysica Acta (BBA) - Reviews on Biomembranes. 1985;822: 355–374. doi:10.1016/0304-4157(85)90015-2

2. Chafai A, Fromm MF, König J, Maas R. The prognostic biomarker L-homoarginine is a substrate of the cationic amino acid transporters CAT1, CAT2A and CAT2B. Sci Rep. 2017;7: 4767. doi:10.1038/s41598-017-04965-2

3. Preston JE, Segal MB, Walley GJ, Zlokovic B V. Neutral amino acid uptake by the isolated perfused sheep choroid plexus. J Physiol. 1989;408: 31–43. doi:10.1113/jphysiol.1989.sp017444

4. Van Winkle LJ, Campione AL, Gorman JM. Na+-independent transport of basic and zwitterionic amino acids in mouse blastocysts by a shared system and by processes which distinguish between these substrates. J Biol Chem. 1988;263: 3150–63. Available: http://www.ncbi.nlm.nih.gov/pubmed/3125176

5. Winkle LJ Van, Campione AL, Farrington BH. Development of system B0,+ and a broad-scope Na+-dependent transporter of zwitterionic amino acids in preimplantation mouse conceptuses. Biochim Biophys Acta Biomembr. 1990;1025: 225–233.

6. Sloan JL, Mager S. Cloning and Functional Expression of a Human Na+and Cl−-dependent Neutral and Cationic Amino Acid Transporter B0+. Journal of Biological Chemistry. 1999;274: 23740–23745. doi:10.1074/jbc.274.34.23740

7. Karunakaran S, Umapathy NS, Thangaraju M, Hatanaka T, Itagaki S, Munn DH, et al. Interaction of tryptophan derivatives with SLC6A14 (ATB0,+) reveals the potential of the transporter as a drug target for cancer chemotherapy. Biochemical Journal. 2008;414: 343–355. doi:10.1042/BJ20080622

8. Karunakaran S, Ramachandran S, Coothankandaswamy V, Elangovan S, Babu E, Periyasamy-Thandavan S, et al. SLC6A14 (ATB0,+) Protein, a Highly Concentrative and Broad Specific Amino Acid Transporter, Is a Novel and Effective Drug Target for Treatment of Estrogen Receptor-positive Breast Cancer. Journal of Biological Chemistry. 2011;286: 31830–31838. doi:10.1074/jbc.M111.229518

9. Smith QR, Momma S, Aoyagi M, Rapoport SI. Kinetics of Neutral Amino Acid Transport Across the Blood‐Brain Barrier. J Neurochem. 1987;49: 1651–1658. doi:10.1111/j.1471-4159.1987.tb01039.x

10. Devés R, Chavez P, Boyd CA. Identification of a new transport system (y+L) in human erythrocytes that recognizes lysine and leucine with high affinity. J Physiol. 1992;454: 491–501. Available: http://www.pubmedcentral.nih.gov/articlerender.fcgi?artid=1175616&tool=pmcentrez&rendertype=abstract

11. Taslimifar M, Faltys M, Kurtcuoglu V, Verrey F, Makrides V. Analysis of L-leucine amino acid transporter species activity and gene expression by human blood brain barrier hCMEC/D3 model reveal potential LAT1, LAT4, B0AT2 and y+LAT1 functional cooperation. J Cereb Blood Flow Metab. 2022;42: 90–103. doi:10.1177/0271678X211039593

12. Van Winkle LJ, Campione AL, Gorman JM. Na+-independent transport of basic and zwitterionic amino acids in mouse blastocysts by a shared system and by processes which distinguish between these substrates. J Biol Chem. 1988;263: 3150–63. Available: http://www.ncbi.nlm.nih.gov/pubmed/3125176

13. O’Kane RL, Viña JR, Simpson I, Zaragozá R, Mokashi A, Hawkins RA. Cationic amino acid transport across the blood-brain barrier is mediated exclusively by system y+. American Journal of Physiology - Endocrinology and Metabolism. Schmitt, U. et al. (2012) ‘In vitro P-glycoprotein efflux inhibition by atypical antipsychotics is in vivo nicely reflected by pharmacodynamic but less by pharmacokinetic changes’, Pharmacology Biochemistry and Behavior, 102(2), pp. 312–320. doi: 10.1016/; 2006. pp. E412–E419. doi:https://doi.org/10.1152/ajpendo.00007.2006
