# Supplementary material for: Saturation kinetics and specificity of transporters for L-arginine and asymmetric dimethylarginine (ADMA) at the blood-brain and blood-CSF barriers
Source: PLoS One. 2025 Jun 11;20(6):e0320034. doi: 10.1371/journal.pone.0320034 (PMC12157126; doi:10.1371/journal.pone.0320034)
Supplement: Attachment — Submitted filename: Response to reviewers.docx [file pone.0320034.s006.docx]

**PONE-D-25-07610**

**Saturation kinetics and specificity of transporters for L-arginine and asymmetric dimethylarginine (ADMA) at the blood-brain and blood-CSF barriers.**

**Journal requirements**

**When submitting your revision, we need you to address these additional requirements.**

**1. Please ensure that your manuscript meets PLOS ONE's style requirements, including those for file naming. The PLOS ONE style templates can be found at** 
[**https://journals.plos.org/plosone/s/file?id=wjVg/PLOSOne_formatting_sample_main_body.pdf**](https://journals.plos.org/plosone/s/file?id=wjVg/PLOSOne_formatting_sample_main_body.pdf) **and** 
[**https://journals.plos.org/plosone/s/file?id=ba62/PLOSOne_formatting_sample_title_authors_affiliations.pdf**](https://journals.plos.org/plosone/s/file?id=ba62/PLOSOne_formatting_sample_title_authors_affiliations.pdf)

**Response to journal requirements**

Thankyou we have amended the manuscript to conform with journal requirements.

**Response to reviewer comments**

Thank you for taking the time to review and comment on our manuscript. We have carefully considered each point and have adapted the manuscript accordingly. Our responses to each query are detailed below. We have also added 20 references to the manuscript in order to justify the additional comments. These are listed in the appendix.

**Reviewer #1: The manuscript described transporters for L-arginine and asymmetric dimethylarginine (ADMA) at the blood-brain and blood-CSF barriers. Authors showed that [3H]-arginine predominately uses system-y+, and not system-B0,+, to cross both the BBB and the blood-CSF barrier (choroid plexus). [3H]-ADMA transport at the BBB and blood-CSF barrier can be mediated by system-y+. Thus, these findings will be useful for the treatment of CNS diseases. Therefore, the manuscript is not too excellent to be published. In other words, the manuscript is so excellent that it should be published.

Comments
Question (1) What was the ratio of the distributed [^3^H]-L-arginine between the BBB barrier and the CSF barrier?**

**Question (2) What was the ratio of the distributed [^3^H]-ADMA between the BBB barrier and the CSF barrier?**

**Question (3) What was the ratio of the distributed [^14^C]-sucrose between the BBB barrier and the CSF barrier?**

**Response to questions 1, 2 and 3**

We have combined our responses to allow for the integration of important points in the discussion. We have also included two more figures in the supplementary information to further support the discussion. These are S14 and S15 figures.

**In the results Lines 541-557 page 21 we have included a new section. It is as follows:**

**Transport across the BBB versus blood-CSF barrier**

Statistical analysis using unpaired two tailed t-tests confirmed that at 10 minutes the uptake of [^3^H]-arginine into the frontal cortex (54.65±9.21 ml.100g^-1^) was 5.7 times higher than that into the CSF (9.53±2.41 ml.100g^-1^; P<0.001) and that the uptake of [^3^H]-arginine into the capillary endothelial cell enriched pellet (6.47±1.018 ml.100g^-1^) was 39.5 times lower than that into the choroid plexus (255.86±30.41 ml.100g^-1^; P<0.0001)(S14 Fig). All values were corrected for [^14^C]-sucrose.

Statistical analysis using unpaired two tailed t-tests confirmed that at 10 minutes the uptake of [^3^H]-ADMA into the frontal cortex (24.94±3.50 ml.100g^-1^) was not significantly different to that into the CSF (26.95±9.02 ml.100g^-1^; P>0.05) and that the uptake of [^3^H]-ADMA into the capillary endothelial cell enriched pellet (2.10±0.86 ml.100g^-1^) was 124.2 times lower than that into the choroid plexus (260.85±42.45 ml.100g^-1^; P<0.001)(S14 Fig). All values were corrected for [^14^C]-sucrose.

[^14^C]-sucrose distribution into the frontal cortex, CSF, capillary depletion enriched pellet samples and the choroid plexus samples was pooled together from the [^3^H]-arginine and [^3^H]-ADMA perfusions and the values compared using unpaired two tailed t-tests (S15 Fig). The distribution of [^14^C]-sucrose into the frontal cortex (3.06±0.38 ml.100g^-1^) was not significantly different to that measured in the CSF (1.75±0.64 ml.100g^-1^). The distribution of [^14^C]-sucrose into the capillary endothelial cells (0.92±0.28 ml.100g^-1^) was 31.4 times lower than that into the choroid plexus (28.88±4.63 ml.100g^-1^; P<0.001).

**In the discussion Lines 793-833 page 31 we have included a new section. It is as follows:**

In this study, we also compared the uptake of [^3^H]-arginine and [^3^H]-ADMA into the brain with that into the CSF at 10 minutes (S14 and S15 Figs). Interestingly, the uptake of [^3^H]-arginine into the brain was significantly higher than that into the CSF, but the uptake of [^3^H]-ADMA into the brain was not significantly different to that measured in the CSF. In both cases, the accumulation of the radiolabelled test molecule in the choroid plexus was significantly higher than that measured in the cerebral capillary endothelial cell (S14 and S15 Figs).

In *in situ* brain/choroid plexus perfusion studies it is not possible to completely separate uptake across the BBB from that across the blood-CSF barrier, thus the relative contributions of the two routes to the concentration of [^3^H]-arginine or [^3^H]-ADMA in the brain or the CSF is not exactly known. In addition, the situation is complicated by the BBB having a lower permeability and greater surface area than the blood-CSF barrier and the complexity of molecule exchange between the brain interstitial fluid and the CSF and *vice versa* [73–75]. However, consideration of the CSF turnover time in rats and humans of approximately 2 and 8 hours, respectively [76][77], and the 10 minute time frame of this mouse perfusion study, does allow us to assume that the (re-)circulating CSF in the glymphatic pathway [1,78] is not the source of the test molecules in the brain parenchyma in these experiments.

Examination of the [^3^H]-arginine, [^3^H]-ADMA and [^14^C]-sucrose accumulation in the different brain regions and compartments, allows us to conclude that [^3^H]-arginine and [^3^H]-ADMA are able to cross the BBB and reach the brain and from there possibly diffuse into the CSF. In addition, direct entry of [^3^H]-arginine and [^3^H]-ADMA into the CSF via the blood-CSF barrier cannot be ruled out. However, the blood-CSF barrier route alone would be unlikely to cause the observed higher concentration of [^3^H]-arginine in the brain than the CSF or the observed homogeneous [^3^H]-ADMA activity between the brain and CSF. This conclusion is reached because the diffusion kinetics from the ventricular CSF into the brain, is considered slower than the rate of bulk flow of CSF out of the ventricles [79]. Thus, the CSF is more likely to act as a 'sink' to the brain than the brain acting as a 'sink' for the ventricular CSF. In addition, even if CSF concentrations exceed brain ISF concentrations and diffusion occurs into the brain ISF, high solute levels may be produced at the ependymal-CSF surface, but as you would expect from a diffusion driven process, there will be an exponential decrease in tissue concentrations at distances as small as a few millimetres from the ventricular surface [79]. Neural cell accumulation would also contribute to this decrease in tissue concentrations. We have previously shown that there was no difference in the distribution of [^3^H]-arginine or [^3^H]-ADMA into the different brain regions including frontal cortex, occipital cortex, hypothalamus, pons, caudate nucleus, hippocampus, thalamus and cerebellum [16]. To conclude, molecules can move from CSF to brain ISF, but compared to the movement across the BBB where diffusional distances between brain capillaries and thus the brain cells is very small, it is very inefficient for reaching large volumes of brain quickly.

The fact that the [^3^H]-ADMA has a similar concentration in the brain and CSF, whereas [^3^H]-arginine has a higher concentration in the brain than the CSF, at the 10 minute perfusion time point, may reflect the removal of [^3^H]-ADMA from the brain tissue into the CSF. In fact, brain to CSF flux and subsequent choroid plexus to blood efflux and/or CSF drainage may contribute to the CNS efflux of ADMA observed in our earlier study [16]. These differences in the handling of [^3^H]-arginine (mol. wt 174.2 g/mol) and the more lipophilic, [^3^H]-ADMA (mol. wt. 202.26 g/mol), by the blood-CNS interfaces must be related to differences in brain cell accumulation and/or transporter interaction, which we observed in our present study.

**New figures in supplementary file.**

The new figures and figure legends are as follows:

**S14 Fig: Comparison of [^3^H]-arginine and [^3^H]-ADMA distribution into frontal cortex (FC), CSF, capillary endothelial cell enriched pellet and choroid plexus (CP).** Uptake is expressed as the percentage ratio of tissue to plasma (mL.100 g^-1^) and is corrected for [^14^C]-sucrose. Perfusion time is 10 minutes. Each bar represents the mean ± SEM of 5-7 animals. Each marker represents one animal. Asterisks represent unpaired t-tests **p* < 0.05, ***p* < 0.01, ****p* < 0.001 (GraphPad Prism 10.0 for Windows).

**S15 Fig: Comparison of [^14^C]-sucrose into frontal cortex (FC), CSF, capillary endothelial cell enriched pellet and choroid plexus (CP).** Uptake is expressed as the percentage ratio of tissue to plasma (mL.100 g^-1^). Perfusion time is 10 minutes. Each bar represents the mean ± SEM of 8-11 animals. Each marker represents one animal. Asterisks represent unpaired t-tests **p* < 0.05, ***p* < 0.01, ****p* < 0.001 (GraphPad Prism 10.0 for Windows).

**(4) Can drug delivery mediated by system-y+ across the BBB be possible using L-arginine or ADMA?**

**(5) Can drug delivery mediated by system-y+ across the CSF be possible using L-arginine or ADMA?**

**Response to questions 4 and 5**

We have combined our responses to allow for the integration of important points in the discussion.

**In the discussion Lines 834-844 page 33 we now state:**

There is substantial evidence that indicates that drugs use transporters to get into and out of cells rather than rely on passive diffusion to cross the lipid bilayer of the plasma membrane [78,79]. For example, system y^+^ is thought to be able to transport the CAA analogue, eflornithine, which is an anti-human African trypanosomiasis drug [28], and system B^0,+^ is thought to transport the cationic anti-influenza compounds, amantadine and rimantadine [27], across the BBB . Prodrugs of non-steroidal anti-inflammatory drugs have also been successfully developed that exploit the endogenous substrate of the LAT transporter to efficiently deliver the prodrug across the BBB [80]. Interestingly, it has recently been suggested that CAT1 may transport large molecules across the BBB [81] and it may be that AAPB and its metabolites (including exogenous arginine) can enter the brain using this transporter. Another important point is that some cancer-causing viruses also use CAA transporters to get into the brain [82,83]. As mentioned previously, molecules (including drugs) are able to cross the BBB and/or the blood-CSF barrier, but would be unlikely to reach deep brain sites if CNS delivery is solely via the blood-CSF barrier.

**(6) What features do system y+ has?**

**Response to question 6**

**We describe the features of system y^+^ on line 613.**

**We have now added an additional point regarding trans-stimulation (in red font).**

**As stated: Line 613-622:**

In this present study we found that [^3^H]-arginine transport into all CNS regions (except CSF) was significantly inhibited (up to 99.7%) by *L*-homoarginine. This indicates that [^3^H]-arginine is entering into the CNS by system y^+^. This is because *L*-homoarginine is a substrate for system y^+^ [36] and the associated transporter proteins i.e. the cationic amino acid transporter-1 (CAT1; SLC7A1), CAT2A (SLC7A2) and CAT2B (SLC7A3) [37]. In addition, CAT1 is expressed at the BBB and the choroid plexus of the blood-CSF barrier [31][38][39][40]. Functionally, CAT1 is a bidirectional uniporter which facilitates the movement of CAAs down a concentration gradient and is usually involved in loading cells with amino acids [41]. Interestingly, system-y^+^ exhibits trans-stimulation, whereby transport is faster when substrate is present on the opposite (*trans-*) side of the membrane [42,43]. System y^+^ activity has been demonstrated on both the luminal and abluminal plasma membranes of the bovine cerebral capillary endothelium, the site of the BBB [33].

**(7) What features do system B0,+, has?**

**Response to question 7**

**As stated lines 627-629:**

BCH and α-methyl-*D,L*-tryptophan are system B^0,+^ inhibitors, which can inhibit CAA (arginine) and neutral amino acid (leucine) transport by the system B^0,+^ transporter protein, ATB^0,+^ (SLC6A14) when expressed in xenopus oocytes or breast cancer cells [44][45] [46].

**As stated lines 672-675**

We, and others, have previously identified the presence of ATB^0,+^ in human BBB cells (hCMEC/D3) using Western blotting and immunofluorescence [15][28][27]. It is thought to be a sodium-dependent symporter involved in the uptake of amino acids into BBB cells (hCMEC/D3) [27,41] [15][28].

**(8) What features do system y+L has?**

**Response to question 8**

**As stated lines 697-706:**

System y^+^L is a heterodimer formed by the interaction of the heavy chain of the cell surface antigen 4F2 (4F2hc, encoded by the SLC3A2gene) with the light chains 4F2-lc2 (or y^+^LAT-1, encoded by SLC7A7) or 4F2-lc3 (or y^+^LAT-2, encoded by SLC7A6). System y^+^L transports CAAs with no need for extracellular Na^+^; however, it can transport both small and large neutral amino acids with high affinity in the presence of this cation [58]. In fact, system y^+^L transporters such as y^+^LAT1 and y^+^LAT2 are thought to exchange a neutral amino acid coupled to a sodium ion for a CAA. System y^+^L transporters are expressed at the human BBB e,g. y^+^LAT1 (SLC7A7) and y^+^LAT2 (SLC7A6) [4]. SLC7A7, SLC7A6 and SLC3A2 mRNA has also been detected in human choroid plexus [39,59][60][61]. In the presence of a CAA loader, CAT1, y^+^LAT1 mediates efflux of CAAs in exchange for neutral amino acids and so these amino acid transporters exhibit functional co-operation.

**(9) What features do system b0,+ has?**

**Response to question 9**

**As stated lines 711-714:**

System b^0,+^ is a facilitated transporter of CAAs, which is inhibited by the organic cation, harmaline [33]. It transports cationic and zwitterionic amino acids. The transporter protein associated with system b^0,+^ is b^0,+^AT and is encoded by the SLC7A9 gene. b^0,+^AT mRNA is expressed in the human brain capillary endothelial cells, but at low levels [4] and b^0,+^AT mRNA was not found in human choroid plexus samples [62].

**Q10 Reviewer #2:** The authors present a carefully undertaken biochemical study informing carrier systems and their contribution to the transport of arginine and ADMA transported across blood-brain and blood-CSF barriers. The study is valuable and informative and deserves publication. The re-use of previously obtained data is stated and rationalized under the view of ethical frameworks. However the value for clarifying the arginine-paradox and its contribution within disorder context seems a bit of an overstatement. Further comments below.

**Response to question 10**

We have now explained the relevance of our study to the arginine paradox in more detail.

In particular we have added further information in the introduction, discussion and conclusion as follows:

**Introduction As stated lines 117:**

In addition, identification of the transporter(s) involved in the delivery of arginine and ADMA into the CNS, may allow us to explain the mechanism behind the arginine paradox, whereby nutritional supplementation with arginine increases NO production despite the intracellular NOS being saturated with arginine [17]. Thus, the observed benefit of arginine supplementation cannot simply be explained by an increase in arginine delivery into the cell, another mechanism of action must be involved.

**Discussion lines 769-780. New explanation in red font.**

As the intracellular concentration of ADMA is higher (e.g. 3.6±1.0 μM rat) than the extracellular concentration (0.33±0.10 μM rat) [65] and the y^+^-system operates by moving cationic amino acids down their concentration gradient so in this case from inside to the outside of cells. It is likely that arginine supplementation would increase NO production by trans-stimulating ADMA efflux rather than inhibiting ADMA influx. Thus, transporter interaction causing a reduction in the intracellular ADMA concentration could explain the arginine paradox, whereby nutritional supplementation with arginine increases NO production despite the intracellular NOS being saturated with arginine. Although other mechanisms possibly in addition to transporter interaction, such as changes to ADMA catabolism [64] and NOS affinity [25][72], cannot be ruled out. Interestingly, normally ADMA concentrations within cells will only inhibit NO production by 10%, but this can increase to 70% when plasma ADMA concentrations are higher than normal [63]. Therefore, arginine supplementation may only be significantly beneficial when ADMA concentrations are elevated above the normal range.

**Conclusion Line 860 we now state:**

This present study identified two transport systems for ADMA (i.e. system y^+^ and system y^+^L) which are involved in ADMA removal from cells and could explain the potential mechanism behind the arginine paradox. We consider that the positive effects of arginine supplementation are due to changes in intracellular ADMA concentrations in part as a direct result of transporter interaction (likely system y^+^).

**Q11 Abstract:
Why does the involvement of several transport systems for ADMA indicate that it is tightly controlled (in comparison to Arginine). Please extend on this statement (best within discussion).**

**Response to question 11 L743-747**

The fact that several CAA transport systems are able to transport ADMA across plasma membranes suggests that its intracellular concentration is flexibly regulated within set limits by multiple pathways [61]. Select transport pathways may be active or inactive simultaneously allowing cells to adapt to changing local conditions. Other mechanisms, such as ADMA catabolism via dimethylarginine dimethylaminohydrolase, may also affect intracellular ADMA concentration [62]. Multiple pathways may be expected due to the importance of ADMA to NO bioavailability.

**Q12: Line 59 partly ? please complete the information. What do you mean by partly, which are the other mechanisms involved.**

**Response to question 12**

**We have now explained the other mechanisms as follows: Lines 59-63**

The selectivity of the barriers is due to the presence of tight junctions, which physically restrict passage between the cells, and the presence of transporters (also called carriers),ion channels and transcytosis machinery, that move substances into and out of the CNS[1]. The barriers also control molecule passage into the CNS by serving as a metabolic (enzymatic) interface.

**Q13 Introduction:
Is generally clear and introduces the background, aim and approach of the study. The last sentence is complex and unclear how these contributions to the field will be achieved by the study. Please rewrite.**

**Response to question 13**

**This last sentence has now been expanded and rewritten as two sentences and is as follows: L114-L120**

This knowledge is essential, not only for understanding the local and neuronal physiological processes that are mediated by the CAAs these carriers transport, but also due to the widespread influence of NO in physiological pathways in health and disease. In addition, identification of the transporter(s) involved in the delivery of arginine and ADMA into the CNS, may allow us to explain the mechanism behind the arginine paradox, whereby nutritional supplementation with arginine increases NO production despite the intracellular NOS being saturated with arginine [17].

**Q14 Methods:**

Line 113 same timeframe ? do you mean one batch of experiment? Please clarify.

**Response to question 14**

**Line 141 now states:**

It is noted that all the raw data was obtained during a period of three years by the same researcher.

**Q15
Please describe the statistical tests used in the paragraph statistics, how where they chosen and if assumptions (normality, equal variance) where tested and how.**

**Response to question 15**

**Lines 325-328 In the statistics section we have now added the following information**

One-way ANOVA (Gaussian distribution assumed and equal SD not assumed) followed by Dunnett's post-hoc test was used to compare the means of the control group to the test group. The results of the post-hoc test were reported. Unpaired two tailed t-tests (Gaussian distribution and equal SD assumed) was used to compare two groups.

**Q16 Results:
Presentation of results is coherent and complete. Figures are clear and provide information of individual data points and sample size.**

**Response to question 16**

Thank you.

**Q17 Discussion:

Paragraph 1-2 are a repetition/extension of the Introduction. This is redundant and not necessary. Paragraph 3 summary of main findings can also be more concise but would be a good paragraph to start the discussion.**

**Response to question 17**

We have removed the first two paragraphs in the discussion starting the discussion with paragraph 3. We have used information in paragraph 1 and 2 to help explain some more points in the introduction.

**Q18 In the abstract and in the conclusion the importance of the presented data for explaining the arginine paradox is stressed. However, I find the link weak, please explain the paradox in more detail and how exactly the data contributes to solving it more clearly.**

**Response to question 18**

**Please see response to question 10**

**Introduction Line 117 we now state:**

In addition, identification of the transporter(s) involved in the delivery of arginine and ADMA into the CNS, may allow us to explain the mechanism behind the arginine paradox, whereby nutritional supplementation with arginine increases NO production despite the intracellular NOS being saturated with arginine [17].

**Discussion lines 769-780. New explanation in red font:**

As the intracellular concentration of ADMA is higher (e.g. 3.6±1.0 μM rat) than the extracellular concentration (0.33±0.10 μM rat) [65] and the y^+^-system operates by moving cationic amino acids down their concentration gradient so in this case from inside to the outside of cells. It is likely that arginine supplementation would trans-stimulate ADMA efflux. Thus, transporter interaction causing a reduction in the intracellular ADMA concentration could explain the arginine paradox, whereby nutritional supplementation with arginine increases NO production despite the intracellular NOS being saturated with arginine. Although other mechanisms possibly in addition to transporter interaction, such as changes to ADMA catabolism [64] and NOS affinity [25][72], cannot be ruled out. Interestingly, normally ADMA concentrations within cells will only inhibit NO production by 10%, but this can increase to 70% when plasma ADMA concentrations are higher than normal [63]. Therefore, arginine supplementation may only be significantly beneficial when ADMA concentrations are elevated above the normal range.

**Conclusion Line 860 we now state:**

This present study identified two transport systems for ADMA (i.e. system y^+^ and system y^+^L) which are involved in ADMA removal from cells and could explain the potential mechanism behind the arginine paradox. We consider that the positive effects of arginine supplementation are due to changes in intracellular ADMA concentrations in part as a direct result of transporter interaction (likely system y^+^).

**Appendix**

**A list of the additional references added to the manuscript to help further clarify points raised by the reviewers.**

1. Divecha YA, Rampes S, Tromp S, Boyanova ST, Fleckney A, Fidanboylu M, et al. The microcirculation, the blood-brain barrier and the neurovascular unit in health and Alzheimer’s Disease. The aberrant pericyte is a central player. Pharmacol Rev. 2025; 100052. doi:10.1016/j.pharmr.2025.100052
2. Gonzalez M, Clayton S, Wauson E, Christian D, Tran Q-K. Promotion of nitric oxide production: mechanisms, strategies, and possibilities. Front Physiol. 2025;16. doi:10.3389/fphys.2025.1545044
3. Ong PK, Moreira AS, Daniel-Ribeiro CT, Frangos JA, Carvalho LJM. Reversal of cerebrovascular constriction in experimental cerebral malaria by L-arginine. Sci Rep. 2018;8: 15957. doi:10.1038/s41598-018-34249-2
4. Moreira AS, Estato V, Malvar DC, Sanches GS, Gomes F, Tibirica E, et al. L-arginine supplementation and thromboxane synthase inhibition increases cerebral blood flow in experimental cerebral malaria. Sci Rep. 2019;9: 13621. doi:10.1038/s41598-019-49855-x
5. Terpolilli NA, Moskowitz MA, Plesnila N. Nitric Oxide: Considerations for the Treatment of Ischemic Stroke. Journal of Cerebral Blood Flow & Metabolism. 2012;32: 1332–1346. doi:10.1038/jcbfm.2012.12
6. Lyck R, Ruderisch N, Moll AG, Steiner O, Cohen CD, Engelhardt B, et al. Culture-Induced Changes in Blood—Brain Barrier Transcriptome: Implications for Amino-Acid Transporters *in vivo*. Journal of Cerebral Blood Flow & Metabolism. 2009;29: 1491–1502. doi:10.1038/jcbfm.2009.72
7. Dahlin A, Royall J, Hohmann JG, Wang J. Expression Profiling of the Solute Carrier Gene Family in the Mouse Brain. Journal of Pharmacology and Experimental Therapeutics. 2009;329: 558–570. doi:10.1124/jpet.108.149831
8. Zani BG, Bohlen HG. Transport of extracellular <scp>l</scp> -arginine via cationic amino acid transporter is required during in vivo endothelial nitric oxide production. American Journal of Physiology-Heart and Circulatory Physiology. 2005;289: H1381–H1390. doi:10.1152/ajpheart.01231.2004
9. Bouldin TW, Krigman MR. Differential permeability of cerebral capillary and choroid plexus to lanthanum ion. Brain Res. 1975;99: 444–448. doi:10.1016/0006-8993(75)90053-0
10. Keep RF, Jones HC. A morphometric study on the development of the lateral ventricle choroid plexus, choroid plexus capillaries and ventricular ependyma in the rat. Developmental Brain Research. 1990;56: 47–53. doi:10.1016/0165-3806(90)90163-S
11. Segal MB. The Choroid Plexuses and the Barriers Between the Blood and the Cerebrospinal Fluid. . Cell Mol Neurobiol. 2000;20: 183–196. doi:10.1023/A:1007045605751
12. Johanson CE. The Choroid Plexus—CSF Nexus. Neuroscience in Medicine. Totowa, NJ: Humana Press; 2003. pp. 165–195. doi:10.1007/978-1-59259-371-2_8
13. Preston JE. Ageing choroid plexus-cerebrospinal fluid system. Microsc Res Tech. 2001;52: 31–37. doi:10.1002/1097-0029(20010101)52:1<31::AID-JEMT5>3.0.CO;2-T
14. Smets NG, Strijkers GJ, Vinje V, Bakker ENTP. Cerebrospinal fluid turnover as a driver of brain clearance. NMR Biomed. 2024;37. doi:10.1002/nbm.5029
15. Collins JM, Dedrick RL. Distributed model for drug delivery to CSF and brain tissue. American Journal of Physiology-Regulatory, Integrative and Comparative Physiology. 1983;245: R303–R310. doi:10.1152/ajpregu.1983.245.3.R303
16. Kell DB, Dobson PD, Bilsland E, Oliver SG. The promiscuous binding of pharmaceutical drugs and their transporter-mediated uptake into cells: what we (need to) know and how we can do so. Drug Discov Today. 2013;18: 218–239. doi:10.1016/j.drudis.2012.11.008
17. Kell DB. The Transporter-Mediated Cellular Uptake and Efflux of Pharmaceutical Drugs and Biotechnology Products: How and Why Phospholipid Bilayer Transport Is Negligible in Real Biomembranes. Molecules. 2021;26: 5629. doi:10.3390/molecules26185629
18. Montaser AB, Järvinen J, Löffler S, Huttunen J, Auriola S, Lehtonen M, et al. L-Type Amino Acid Transporter 1 Enables the Efficient Brain Delivery of Small-Sized Prodrug across the Blood–Brain Barrier and into Human and Mouse Brain Parenchymal Cells. ACS Chem Neurosci. 2020;11: 4301–4315. doi:10.1021/acschemneuro.0c00564
19. Kurtyka M, Wessely F, Bau S, Ifie E, He L, de Wit NM, et al. The solute carrier SLC7A1 may act as a protein transporter at the blood-brain barrier. Eur J Cell Biol. 2024;103: 151406. doi:10.1016/j.ejcb.2024.151406
20. Shin S, Thapa SK, Fung H-L. Cellular interactions between L-arginine and asymmetric dimethylarginine: Transport and metabolism. PLoS One. 2017;12: e0178710. doi:10.1371/journal.pone.0178710
